# Differences in Aerobic Fitness between Inpatients and Outpatients with Severe Mental Disorders

**DOI:** 10.3389/fpsyt.2014.00095

**Published:** 2014-08-04

**Authors:** Helene Daae-Qvale Holmemo, John Christian Fløvig, Jørn Heggelund, Einar Vedul-Kjelsås

**Affiliations:** ^1^Faculty of Medicine, Norwegian University of Science and Technology, Trondheim, Norway; ^2^Department of Neuroscience, Faculty of Medicine, Norwegian University of Science and Technology, Trondheim, Norway; ^3^Division of Psychiatry, Department of Østmarka, St. Olavs University Hospital, Trondheim, Norway; ^4^Department of Research and Development (AFFU), Division of Psychiatry, St. Olavs University Hospital, Trondheim, Norway

**Keywords:** aerobic fitness, inpatients, outpatients, mental disorders, VO_2_max, psychosis, cardiovascular risk factors, mortality

## Abstract

**Background:** Patients with severe mental disorders have increased mortality, and cardiovascular disease (CVD) accounts for a large part. Physical inactivity and low aerobic fitness have been recognized as significant risk factors for CVD. In this study, we investigated the differences in aerobic fitness and physical activity between in- and outpatients with severe mental disorders.

**Method and Subjects:** Fifty in- and outpatients from a regional psychiatric department were included. The patients filled in a questionnaire on physical activity and completed a clinical examination. An estimation of aerobic fitness was calculated for each patient, using gender, age, waist circumference, resting heart rate, and physical activity level as variables.

**Results:** Inpatients had lower estimated aerobic fitness than outpatients (VO_2_peak 42 vs. 50 mL kg^−1^ min^−1^, *p* < 0.001). Compared to population data matched for age and gender, inpatients had lower aerobic fitness, while outpatients were not different from the population average.

**Conclusion:** Inpatients at a psychiatric department had lower estimated aerobic fitness than outpatients, and a lower aerobic fitness compared to the general population. Our findings suggest that inpatients with severe mental disorders should be considered a high risk group for CVD.

## Introduction

Patients suffering from severe mental disorders have higher mortality rates than the general population (1, 2). The excess mortality is mainly caused by somatic disease and cardiovascular disease (CVD) accounts for a large part (3, 4). Physical inactivity is a risk factor for CVD, and reduced aerobic fitness is a predictor for CVD and all-cause mortality (5). Patients with mental disorders have low levels of physical activity and low aerobic fitness compared to the general population (6, 7), and Heggelund et al. (8) found that subjects with low aerobic fitness had an increased risk of having traditional CVD risk factors. Despite being an important risk factor for CVD, there are few studies measuring aerobic fitness in patients with severe mental disorder. Most studies have only included patients with schizophrenia.

A recent study from Finland indicated that there is a difference in mortality between inpatients and outpatients with schizophrenia (9). Inpatients presumably have more severe symptoms than outpatients, thus representing a different patient group than outpatients. However, we do not know if there are differences between inpatients and outpatients when it comes to physical activity and aerobic fitness. We found no previous studies where aerobic fitness in inpatients and outpatients with severe mental disorders was compared.

We hypothesized that both in- and outpatients had lower aerobic fitness compared to a population average, and that inpatients had lower aerobic fitness than outpatients.

## Materials and Methods

### Study population

All patients in the psychosis section at Østmarka psychiatric department (St. Olavs Hospital, Trondheim, Norway) were asked to participate. Østmarka psychiatric department is a public, specialized hospital for patients with severe mental disorders. The department is serving a population of approximately 300,000 inhabitants in the county of Sør-Trøndelag in the middle of Norway, with mixed urban and rural areas. Patients are referred to the department when extensive resources for treatment and diagnosis are needed. Patients admitted to the psychosis section have suspected or known psychosis, severe affective disorder or other disorders requiring long-term psychiatric rehabilitation. A large part of the patients have comorbid or complicating mental disorders, including substance abuse. The section has three inpatient units, an outpatient clinic, and an exercise training clinic available for both in- and outpatients. Patients only attending the exercise training clinic were not asked to participate.

#### Inclusion criteria

Inpatients in the psychosis section between the 23rd of September and the 1st of November 2013 or outpatients attending a consultation during the same period.

#### Exclusion criteria

Patients who could not understand Norwegian language and patients who were not able to complete the test procedures.

The patients were asked for informed, written consent. A total of 86 patients were identified for inclusion, 48 inpatients and 38 outpatients. One patient was excluded because of language problems, 4 were unavailable for request, and 31 patients did not want to participate. A total number of 50 patients gave their consent to participate in the study.

### Procedure

Each patient completed a questionnaire on physical activity, lifestyle, dietary habits, and demographic data. We used the same questions on these topics as the HUNT-study (The Nord-Trøndelag Health Survey) (10). The HUNT study is a large population based health study in Norway, carried out in three waves between 1984 and 2008 in the county of Nord-Trøndelag, Norway.

The patients were clinically examined, including measurement of blood pressure (BP), resting heart rate (RHR), height, weight, and waist circumference (WC). BP was measured using an automatic BP device (OSZ5 easy, Welch Allyn). RHR was measured using a heart rate monitor (Polar RS400) after at least 5 min of rest in a calm environment. WC was measured at umbilicus level, using a Myotape measuring tape. Weight (Seca 888) was measured while wearing light clothing and no shoes. Height and weight was read to the nearest centimeter and kilogram, respectively. Body mass index (BMI) was calculated (weight/height^2^).

Peak oxygen uptake [VO_2_peak (mL kg^−1^ min^−1^)] is considered the most valid measure of aerobic fitness (11). This is done by analyzing ventilation gas during strenuous aerobic exercise, which requires expensive equipment, skilled personnel and patients willing to participate in a demanding physical test. However, VO_2_peak can be estimated from non-exercise parameters (12). The method has been found to be reasonably accurate compared to direct measurement in a large population sample. A recent study (13) also states that estimated VO_2_peak predicts long-term risk of premature CVD mortality and all-cause mortality with accuracy similar to what has been obtained using a direct measure of VO_2_peak.

The physical activity for each patient was measured by a physical activity index (PA-index), as according to Nes et al. (12). PA-index is calculated by multiplying the scores for frequency, intensity, and duration of physical activity, based on the patients’ self report on physical activity.

Estimated VO_2_peak was calculated using the formulas described by Nes et al. (12):

$$\begin{array}{llll} \text{V}{\text{O}}_{\text{2}}\text{pea}{\text{k}}_{\text{Female}} & =74.74-\left(0.247\times \text{age}\right)-\left(0.259\times \text{WC}\right)\; & & \;\\ & \;-\left(0.114\times \text{RHR}\right)+\left(0.198\times \text{PA}-\text{index}\right)\; & & \;\\ \text{V}{\text{O}}_{\text{2}}\text{pea}{\text{k}}_{\text{Male}} & =100.27-\left(0.269\times \text{age}\right)-\left(0.369\times \text{WC}\right)\; & & \;\\ & \;-\left(0.115\times \text{RHR}\right)+\left(0.226\times \text{PA}-\text{index}\right)\; & & \; \end{array}$$

A fasting blood sample was collected and total cholesterol, HDL cholesterol, estimated LDL-cholesterol, triglycerides, and fasting glucose were analyzed.

Traditional risk factors for CVD were classified as follows (14–16): smokers: patients who reported to smoke daily or occasionally. Hypertension: systolic BP ≥ 140 mmHg and/or diastolic BP ≥ 90mmHg. Elevated total cholesterol: >6.1 mmol/L in patients younger than 30 years, >6.9 mmol/L inpatients from 30 to 49 years, and >7.8 mmol/L in patients 50 years or older. Elevated LDL-cholesterol: >4.3 mmol/L, >4.7 mmol/L, and >5.3 mmol/L in the same age groups, respectively. Low HDL cholesterol: <1 mmol/L. Elevated triglycerides: >2.6 mmol/L. Hyperglycemia: glucose >6 mmol/L. Obesity: BMI ≥ 30 kg/m^2^.

Diagnosis and use of medication were collected from the patients’ medical records. For patients who had not received a formal diagnose at the end of the inclusion period, the patients’ psychiatrist or psychologist was asked for diagnosis, which was also reviewed by the second author. The total number of days the patients’ had been admitted to psychiatric hospital was counted in the medical record. Both admissions to the current institution and admissions to other psychiatric hospitals were counted.

Aspenes et al. (17) have measured VO_2_peak directly in more than 4500 people in the HUNT study and calculated the average VO_2_peak for specified groups based on age and gender. We used these averages to compare the estimated VO_2_max for each patient with the average for the corresponding age and gender.

### Statistical analysis

For statistical analysis, we used IBM SPSS Statistics v.21. Data are described using mean and standard deviation (SD) unless otherwise noted. Independent sample *T*-test was used for continuous variables. Pearson’s Chi-square test or Fisher’s exact test were used for categorical variables.

### Ethics

The study was approved by The Regional Committee for Medical and Health Research Ethics (REK) and The Norwegian social science data services (NSD). The study was conducted according to the Helsinki declaration.

## Results

A total of 23 inpatients and 27 outpatients participated in the study. All 50 patients filled in the questionnaire and completed the physical measurements. A fasting blood sample was collected from 49 patients. A description of the study population is presented in Table 1.

**Table 1:** **Description of the study population**.

Variable	Total	Inpatient	Outpatient	Statistics^a^
Description of patients
*N*	50	23	27	–
Age (years), mean ± SD	29.5 ± 11.1	31.5 ± 13.5	27.8 ± 8.5	NS
Female *n* (%)	16 (32)	10 (43)	6 (22)	NS
Coercion	13 (26)	13 (57)	0 (0)	*p* < 0.001
No work or daily activity, *n* (%)	26 (52)	18 (78)	8 (30)	*p* < 0.001
Lives alone, *n* (%)	23 (46)	13 (57)	10 (37)	NS
Psychosis (F20–29), *n* (%)	27 (54)	15 (65)	12 (44)	NS
Affective (F30–39), *n* (%)	13 (26)	5 (22)	8 (30)	NS
Other diagnoses	10	3 (13)	7 (26)	NS
Physical measurements, mean ± SD
Height (cm)	178 ± 0.1	178 ± 0.1	178 ± 0.1	NS
Weight (kg)	82 ± 20.2	83 ± 17.8	81 ± 22.3	NS
Waist circumference (cm)	89 ± 16.0	92 ± 15.3	86 ± 16.3	NS
Resting heart rate (bpm)	76 ± 14.3	81 ± 15.5	73 ± 12.3	NS

*^a^NS, not significant*.

Inpatients had more inpatient days in psychiatric treatment than outpatients over the last 2 years (168 vs. 30 days, *p* = 0.003) and all life (356 vs. 71 days, *p* = 0.021). More inpatients used antipsychotic medication (20 out of 23 vs. 12 out of 27, *p* = 0.002). The mean estimated VO_2_peak for patients using antipsychotic medication was not statistically different from other patients (45 vs. 48 mL kg^−1^ min^−1^, *p* = 0.29). Six out of 23 inpatients and 2 out of 27 outpatients had somatic diseases (heart disease, vascular disease, diabetes, and hypertension). There were no statistically significant differences in somatic disease between the two groups. Data necessary to calculate estimated VO_2_peak and data on height and weight is presented in Table 2. The frequency of risk factors for CVD is presented in Table 3.

**Table 2:** **Physical activity data and measurements for 50 patients with severe mental disorders**.

Physical activity data	All patients (*n* = 50)	Inpatients (*n* = 23)	Outpatients (*n* = 27)	Statistics^a^
Frequency *n* (%)
Inactive	12 (24)	5 (22)	7 (26)	NS
Once a week	2 (4)	2 (9)	0 (0)	NS
2–3 Times a week	25 (50)	10 (43)	15 (55)	NS
Almost every day	11 (22)	6 (26)	5 (19)	NS
Intensity^b^ *n* (%)	*n* = *38*	*n* = *18*	*n* = *20*	
Take it easy	8 (21)	6 (33)	2 (10)	NS
Heavy breath and sweat	23 (61)	11 (61)	12 (60)	NS
Near exhaustion	7 (18)	1 (6)	6 (39)	NS
Duration^b^ *n* (%)	*n* = 38	*n* = 18	*n* = 20	
<15 min	3 (8)	3 (17)	0 (0)	NS
15–29 min	4 (11)	3 (17)	1 (5)	NS
30–60 min	21 (55)	9 (50)	12 (60)	NS
>60 min	10 (26)	3 (17)	7 (35)	NS
Physical measurements, mean ± SD
Height (cm)	178 ± 10.0	178 ± 10.6	178 ± 9.7	NS
Weight (kg)	82 ± 20.2	83 ± 17.8	81 ± 22.3	NS
Waist circumference (cm)	89 ± 16.0	92 ± 15.3	86 ± 16.3	NS
Resting heart rate (bpm)	76 ± 14.3	81 ± 15.5	73 ± 12.3	NS

*^a^NS, not significant (independent sample *T*-test, Chi-square test, or Fisher’s exact test)*.

*^b^Questions on intensity and duration were only addressed to those who reported to exercise at least once a week*.

**Table 3:** **Frequency of traditional risk factors for CVD in 50 patients with severe mental disorders**.

Risk factors	All patients (*n* = 50)	Inpatients (*n* = 23)	Outpatients (*n* = 27)	Statistics^a^
Blood pressure
Systolic (mmHG), mean ± SD	130 ± 18.6	129 ± 15.0	131 ± 17.6	NS
Diastolic (mmHG), mean ± SD	77 ± 16.3	76 ± 13.3	78 ± 10.7	NS
Hypertension *n* (%)	11 (22)	6 (26)	5 (19)	NS
Smoking, *n* (%)
Daily	15 (30)	10 (43)	5 (18)	NS
Occasionally	11 (22)	4 (17)	7 (26)	NS
Total	26 (52)	14 (60)	12 (44)	NS
Obesity
BMI (kg m^−2^), mean ± SD	25.4 ± 6.7	26.3 ± 5.2	25.5 ± 6.0	NS
Obese *n* (%)	11 (22)	7 (30)	4 (15)	NS
Cholesterol^b^	*n* = 49	*n* = 23	*n* = 26	
Total (mmol/L), mean ± SD	4.6 ± 0.90	4.6 ± 0.67	4.6 ± 1.1	NS
HDL (mmol/L), mean ± SD	1.4 ± 0.67	1.2 ± 0.29	1.6 ± 0.85	NS
LDL (mmol/L), mean ± SD	2.8 ± 0.78	2.9 ± 0.64	2.7 ± 0.89	NS
Triglycerides (mmol/L), mean ± SD	1.8 ± 0.68	1.1 ± 0.5	1.2 ± 0.82	NS
High total chol, *n* (%)	2 (4)	0 (0)	2 (8)	NS
High LDL *n* (%)	2 (4)	0 (0)	2 (8)	NS
High triglycerides, *n* (%)	2 (4)	0 (0)	2 (8)	NS
Low HDL *n* (%)	8 (16)	4 (17)	4 (15)	NS
Glucose^b^
Fasting glucose (mmol/L), mean ± SD	5.2 ± 1.0	5.2 ± 1.0	5.1 ± 1.0	NS
Hyperglycemia, *n* (%)	5 (10)	3 (13)	2 (8)	NS

*^a^NS, not significant (independent sample *T*-test, Chi-square test, or Fisher’s exact test)*.

*^b^Blood samples were collected from 49 patients*.

The mean estimated VO_2_peak for inpatients were 42 mL kg^−1^ min^−1^ (SD 10.0) and 50 mL kg^−1^ min^−1^ (SD 8.1) for outpatients. The difference was statistically significant (*p* < 0.001). The mean estimated VO_2_peak for all patients was 46 mL kg^−1^ min^−1^ (SD 9.7).

**Figure 1:**
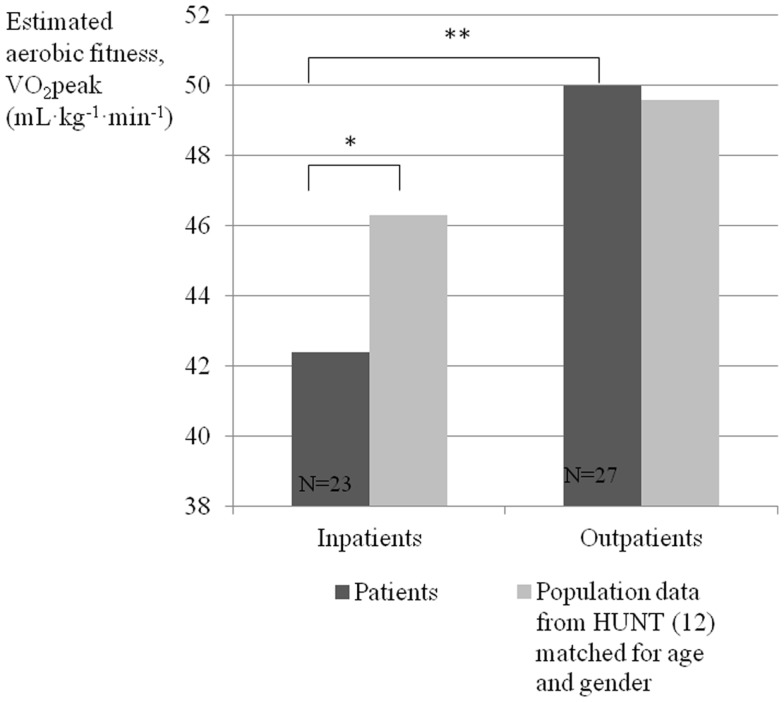
**Estimated VO_2_peak for inpatient and outpatient with severe mental disorder, compared to a matched population average**. *p=0.003. **p < 0.001.

Inpatients had 3.9 mL kg^−1^ min^−1^ lower estimated VO_2_peak than the matched HUNT average (Figure 1). The outpatients had 0.33 mL kg^−1^ min^−1^ higher in absolute terms, but not statistically different (SD5.6, *p* = 0.8). The deviation from matched HUNT average was larger for inpatients than for outpatients (*p* = 0.025).

## Discussion

The main finding in this study is that inpatients with severe mental disorders had a lower level of estimated VO_2_peak than outpatients. This difference was also evident when we compared the groups with a matched population average. The finding of a low VO_2_peak in inpatients is in accordance with previous studies on aerobic fitness in patients with severe mental disorders (8). It fits well with knowledge about a low level of physical activity in this group (6, 7). The low level of aerobic fitness will probably contribute to an increased risk of CVD among inpatients, and thereby highlight inpatients as a high risk group for CVD among patients with severe mental disorders.

The outpatients in our study were not different from the matched population average with respect to estimated aerobic fitness. This was contrary to our hypothesis. We expected to find that outpatients as well as inpatients were below the population average, but our findings indicate that outpatients could have the same level of aerobic fitness as the general population. This could indicate that outpatients have a relatively lower risk for CVD compared to inpatients This may in turn contribute to a difference in mortality the two groups, as found by Kiviniemi et al. (9). The study population is, however, young, due to a considerable number of patients in early phases of their disorder being assessed and diagnosed. Some patients may therefore not yet have experienced the full consequences of the disorders when it comes to lifestyle and risk factors for CVD. The fact that they still might have an average risk of CVD, suggests that it is possible to live with a severe mental disorder and still keep the risk for developing CVD on a population level. A relatively high proportion of the outpatients had work or daily activity (19 out of 27, 70%), and this probably contributes to physical activity and better aerobic fitness.

We found no statistically significant differences between inpatients and outpatients in the factors used to estimate aerobic fitness, even though we found a difference in the resulting estimated fitness. This suggests that there could be differences in the separate factors, including physical activity, that are not revealed in this study because of the small size of the study population.

The number and characteristics of patients admitted to inpatient treatment in a psychiatric hospital will vary in areas, depending on the availability of inpatient treatment and selection of patients. The inpatient group identified in this study might therefore receive other care in other locations, depending on how the health service is organized. Our findings still show that there are groups of patients with severe mental disorders with low aerobic fitness and other groups with normal aerobic fitness that need to be identified in order to treat the individual patient needs.

More inpatients used antipsychotic medication in this study. This is not surprising given the presumption that inpatients have more severe symptoms and need more treatment. There are concerns that antipsychotic medications would increase prevalence of risk factors for CVD, and the effects of medications could also affect heart rate and WC (18) used to estimate VO_2_peak in this study. We did not find a statistically significant difference in estimated aerobic fitness between patients using antipsychotic medication and other patients. This suggests that the effects of antipsychotic medications cannot explain the difference in VO_2_peak between inpatients and outpatients found in this study.

This study has some limitations. Our results are based on an estimation of the VO_2_peak, not a direct measurement as most of the previous studies have been. Some of the variables used in the formulas to estimate the VO_2_peak are subject to measuring error or reporting error, namely, RHR, WC, and PA-index. To account for this, the same equipment was used for all patients, and the same person carried out all the measuring. The PA-index is based on self report, and therefore, comes with some degree of uncertainty. However, the questions used to estimate the PA-index have been found to have high reliability and validity (19). It is also found that the estimated aerobic fitness can predict all-cause mortality and premature cardiovascular death, which supports that it is a valid measure and sufficient for the purpose of this study.

Some of the patients were involuntarily admitted to hospital for psychotic disorders, and therefore, in a particularly vulnerable situation. However, the examinations and questions in this project could cause little or no harm, and possible negative consequences of participating were considered to be insignificant.

## Conclusion

Our results show that inpatients with severe mental disorders have a lower estimated aerobic fitness than outpatients, and that inpatients have a lower average aerobic fitness compared to the general population. This suggests that inpatients with severe mental disorders should be highlighted as a high risk group for CVD.

## Conflict of Interest Statement

The authors declare that the research was conducted in the absence of any commercial or financial relationships that could be construed as a potential conflict of interest.
